# Influence of Composite Thickness on Ultrasonic Guided Wave Propagation for Damage Detection

**DOI:** 10.3390/s22207799

**Published:** 2022-10-14

**Authors:** Tianyi Feng, Zahra Sharif Khodaei, M. H. Ferri Aliabadi

**Affiliations:** Structural Integrity and Health Monitoring, Department of Aeronautics, Imperial College London, South Kensington, London SW7 2AZ, UK

**Keywords:** Structural Health Monitoring (SHM), lead zirconate titanate (PZT) transducers, thickness influence, thick composites, damage detection and localisation

## Abstract

In this paper, the propagation properties of ultrasonic guided waves (UGWs) in different-thickness composites (i.e., 2, 4 and 9 mm) were critically assessed, and their effectiveness for damage detections and localisations under varying temperatures was demonstrated. A diagnostic film with phased-array lead zirconate titanate (PZT) transducers based on the ink-jet printing technique was used in the experiments. Initially, the dispersion curves for these composites were compared. Next, the effects of the composite thickness on the A_0_ and S_0_ mode amplitudes and the group velocity were investigated by active sensing. Next, the behaviours of UGWs under varying temperatures in different-thickness plates were also investigated. Finally, surface-mounted artificial damage and impact damage were detected and located in different composites.

## 1. Introduction

Fibre-reinforced plastic composites are now a prominent material in modern commercial airframe aircraft [1,2]. These types of composites have a superior strength-to-weight ratio, higher tensile strength and stiffness, good properties of corrosion/fatigue resistance and higher thermal stability [1,2,3,4,5]. For instance, the usages of composites are up to 50% by weight content in aircraft structures for Boeing Dreamliner 787 and 52% for Airbus 350 [2]. A key consideration for composites is their susceptibility to low-velocity impact damage, which is not detectable by the naked eye, commonly referred to as barely visible impact damage (BVID) [1,6,7]. The characterisation of BVID in different-thickness composites is complicated and differs for each composite structure, depending on its layup, material and thickness [8]. Failure to detect such damages may lead to unexpected failures, and therefore, their early detection is of great interest within the aeronautics sector [9,10].

The internal defects in composites are complex, consisting of multimode damage and the extent of the impact damage normally spreading across the thickness under the impacted surface. In thin composite structures, matrix cracking caused by impact is generated from the lowest ply layer because of bending stress. In addition, a reverse pine-tree pattern is formed, since intra-ply cracks and interface delamination propagate from the lowest surface to the impact surface [11]. In thick composite structures, the damage propagates away from the impact location in the shape of a cone and forms a pin-tree pattern [11]. It is also demonstrated that thicker composite structures have higher bending stiffness and higher compression after impact strength [8]. In addition, the damage mechanism for thick composite structures is different from that of thin composite structures [8]. Thick composite structures cause larger delamination, while thin composite structures cause more fibre breakage during the low-velocity impact. The reason for the latter is that the fibre breakage dominates, and the effects of matrix cracking can be ignored inducing suppression of delamination [8].

Structural Health Monitoring (SHM) can play an important role in the early detection of BVID. These inspection techniques are developed with the aim of detecting damage, hence allowing the real-time health of the structure (e.g., the size and the location of the damage) and analysing the load history of the aircraft [12,13,14,15,16]. Ultrasonic guided waves (UGWs) actuated by lead zirconate titanate (PZT) transducers are widely used to monitor composite structures due to their low attenuation to propagate long distances [17,18,19]. Generally, UGWs consist of symmetric (Si) and anti-symmetric (Ai) modes with different phases and group velocities [7]. Different modes are excited, depending on the frequency and thickness of the composite, with each having different dispersion and propagation properties, making each suitable for detecting different damage sizes and types [20,21].

For diagnostic purposes based on SHM techniques, UGWs should ideally be non-dispersive, lowly attenuative, and highly sensitive to BVID, which requires an optimum wave mode to be selected [7]. However, this information is not known; therefore, it is important that during the design and development phase of any SHM system, a detailed investigation is carried out to choose the optimum parameters of the UGW (frequency, amplitude and mode), which will interact with probable damage types in the plate and enable a reliable damage detection. Many studies have been numerically carried out to capture the guided wave propagation properties in thin composite structures of different layups, materials, complexities and sizes numerically, since experimental investigations are often timely and expensive to allow a deeper understanding of the wave/damage interaction to optimise the parameters of the SHM system, towards condition-based maintenance concepts [5,11,18,20,21,22,23,24,25,26,27,28,29,30,31,32,33].

However, there are limited studies focused on thick composite structures. James and Giurgiutiu [34] compared the BVID size resulting from different impact energies and forces for coupons with different thicknesses. They have demonstrated that the impact damage propagated through the thickness for the 2 mm and 4 mm thick coupons, but not for the 6 mm thick coupons (thick composites). In addition, the controlled impact size was more difficult to obtain for the thick composite coupon compared to for the thin composite coupon. Moreover, the indentation shape for the thicker composite coupon was flatter than that of the thinner one, given the hemispherical tip of the tup for the impactor. However, their study did not include any guided wave or wave/damage interaction investigation for different-thickness composites. Abetew et al. [35] used a pulse-echo laser ultrasonic system to inspect thick composite structures (thicknesses of 8 mm and 10.3 mm). They found that a larger laser beam size could increase the bigger peak-to-peak amplitude of guided waves which significantly increased the propagation depth of UGWs into the structure and could improve the capability of inspecting thick composite structures.

Furthermore, Duan et al. [8] numerically studied and experimentally validated low-velocity impact tests and frequency-sweep vibration tests for thick composite laminate (5 mm). They showed a good agreement between the simulation and the experiments. They found that the damage occurred at the outer plies first for thick composite laminates due to the fact that the matrix damage was the high contact stress and laminate bending deformation under impact load. The delamination happened at the upper plies and propagated to the inner plies due to high contact stress and formed a pine-type damage pattern in the thickness direction. By increasing the impact energy, a larger damage area was obtained, and the damage shape changed from an ellipse to a “peanut” shape and then to an irregular shape in the end. Andreades et al. [36] proposed a novel nonlinear ultrasonic localisation method for BVID in thick composites (thicknesses of 6 mm and 13 mm). This method did not require signal transmission frequency, the time-of-flight of guided waves, and the need to obtain the baseline state of composites’ structures. Their experiment results showed that the BVID within an error range of 4–22 mm can be successfully located.

However, none of the above studies investigated the UGW propagation (wave modes and propagation velocities) through thick composite structures in detail and under different environmental conditions. Due to the anisotropic behaviour and complex BVID scenarios [22,25] and extremely high attenuation [35] of thick composite structures, it is a challenge to successfully detect damage in thick composite structures based on GW-SHM techniques.

Previously, Bekas et al. [37] developed diagnostic films with PZT transducers based on the ink-jet printing technique that can be used to replace traditional wired cables to reduce 1/3 weight of extra cables. Compared to the Stanford Multi-Actuator Receiver Transduction (SMART) Layer^TM^, the diagnostic film based on the ink-jet printing technique can meet the operational and environmental conditions of the regional aircraft for low-/high- temperature changes under cyclic loading [38], while the SMART Layer^TM^ cannot [39]. In the authors’ previous work [40], the diagnostic films with PZT transducers were embedded and mounted on the surface of thick composite laminates to evaluate and compare the electro-mechanical impedance properties, temperature influence on UGWs, residual guided wave signals between the baseline and damage signals and interactions of UGWs with surface-mounted artificial damage and impact damage using a laser Doppler vibrometer.

In this paper, an in-depth investigation into the influence of the composite thickness on the effectiveness of UGWs under different temperatures is presented for the first time. It has been demonstrated that the composite thickness affected the results of damage detection and localisation. The investigation was informed by the use of theoretical dispersion curves for composites with different thicknesses. UGW propagation in a thick composite plate (9 mm) was compared to that in thin composite plates (2 mm and 4 mm) to highlight the different propagation properties that were thickness-dependent and can influence the reliability of a detection methodology. Furthermore, the temperature influences on guided wave signals through composites with different thicknesses were studied, followed by damage detection and localisation for surface-mounted artificial damage and impact damage.

## 2. Experimental Setup

**Figure 1:**
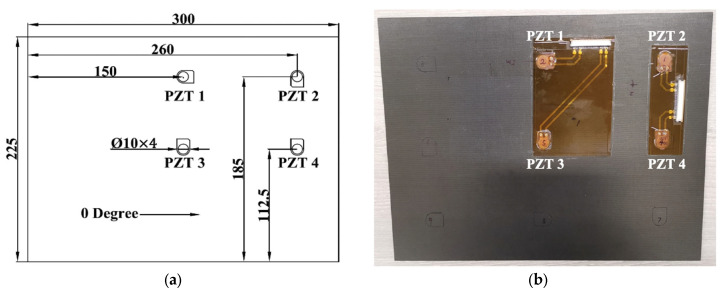
(**a**) Drawing of the specimen; (**b**) example of the composite panel with PZT transducers.

To compare the thickness effect on UGWs, three different composite panels with thicknesses of 2 mm, 4 mm and 9 mm were fabricated. The reason for choosing these numbers was that thicknesses of 2 mm and 4 mm are typical of fuselage skin and the thickness of 9 mm is representative of a composite frame in the fuselage or other high-load-bearing parts on an aircraft wing. Unidirectional carbon fibre prepregs (Hexply^®^ IM7/8552) were chosen, and the average thickness of each cured ply was 0.125 mm. The quasi-isotropic stacking sequence for the lay-up was [(0°/45°/−45°/90°)_n_]_s_, where n was 2, 4 and 9 for each panel, respectively. The general drawing of these panels and locations for each PZT transducer is shown in Figure 1a. After curing, those panels were inspected by a DolphiCam ultrasonic camera.

For preparing the diagnostic films, a Kapton^®^ film (25.4 µm for the thickness, 400 °C for the melting temperature) with printed circuits was used in these experiments, as shown in Figure 1b. A Dimatix printer (DMP-2580) was used to print the circuits, and the nanoparticle concentration for the silver-based ink was 30–35 wt %. To have a stable drop formation of the printing, the piezo voltage was set to 20 V with a 5 kHz jetting frequency for the waveform setup. The drop spacing was 35 μm, and the substrate temperature was set to 55 °C to have an ideal printing quality. To reduce the circuit’s resistance, the width of the circuits was set to 1.4 mm, and the printing was repeated 3 times. The printed Kapton^®^ films were put in an oven to cure at 135 °C for 1 h to make all nanoparticles inks conductive.

After the printed Kapton^®^ films were ready, all soldering points for each PZT transducer and one side of high-temperature connecting terminals (TML Co. TPF-2MS) were applied with small amounts of mixed conductive epoxy/hardener (RS 186-3616). These PZT transducers and connecting terminals were placed at designated positions on the film. To cure the conductive epoxy/hardener, these Kapton^®^ films were subsequently placed into the oven to cure at 80 °C for 20 min. After that, two layers of adhesive films were placed on the designated positions of each panel, followed by Kapton^®^ films with printed circuits and integrated PZT transducers. Finally, these panels were then cured at 150 °C for 1 h with a vacuum until cooling down to room temperature. After bonding, all edges were trimmed by a cutting machine, and two connectors (RS 514-4408) were mounted on the designated edges of each panel bonded by a superglue (RS 473-445). Then, these connecting terminals were soldered to connect to the two connectors of each panel.

## 3. Dispersion Curves

**Figure 2:**
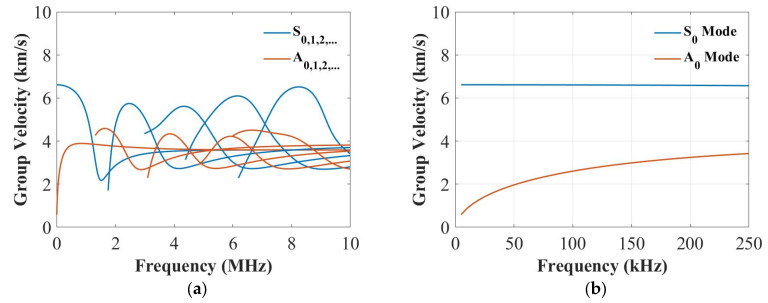
Dispersion curves for the group velocity from 0 to 10 MHz (**a**) and from 0 to 250 kHz (**b**) for the 2 mm thick panel.

**Figure 3:**
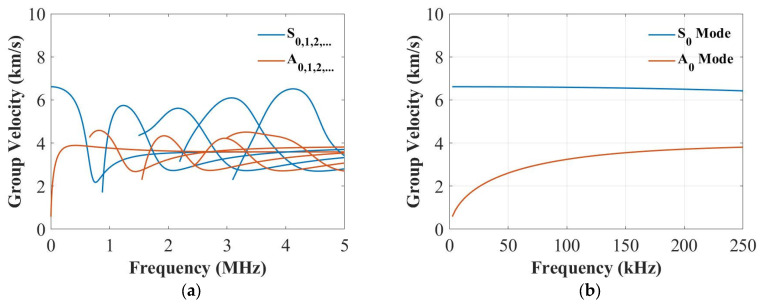
Dispersion curves for the group velocity from 0 to 5 MHz (**a**) and from 0 to 250 kHz (**b**) for the 4 mm thick panel.

**Figure 4:**
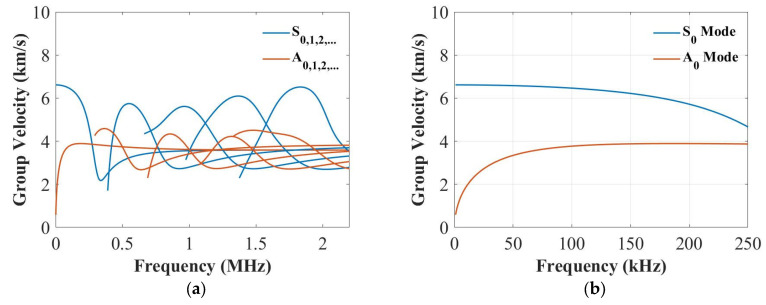
Dispersion curves for the group velocity from 0 to 2.5 MHz (**a**) and from 0 to 250 kHz (**b**) for the 9 mm thick panel.

In this section, the dispersive curves based on the analytical method [41] were plotted to study the thickness influence on the group velocities of the S_0_ and the A_0_ modes. Figure 2, Figure 3 and Figure 4 plot the dispersion curves of quasi-isotropic panels for the 2 mm, 4 mm and 9 mm thick panels at 0°, respectively. According to these figures (Figure 2a, Figure 3a and Figure 4a), the shapes of these curves look similar, and the only difference is their frequency range. Figure 2b, Figure 3b and Figure 4b show the dispersion curves from 0 to 250 kHz, which clearly showed the group velocity of the A_0_ and the S_0_ modes at 250 kHz became closer when increasing the thickness. This means the thickness greatly affected the group velocity of the S_0_ mode at 250 kHz.

## 4. Ultrasonic Guided Waves

In this section, the comparisons of UGWs for all these panels were investigated, and amplitudes of the first wave packets were compared to investigate the thickness influence to develop optimum excitation parameters for damage detection purposes at various plate thicknesses. Since SHM systems utilize point sensors such as PZT actuators, the responses (in-plane) of the guided wave propagation recorded by PZT sensors were investigated in detail, as these results were used for damage detection and characterisation and can be deduced from the recorded full-field response.

**Figure 5:**
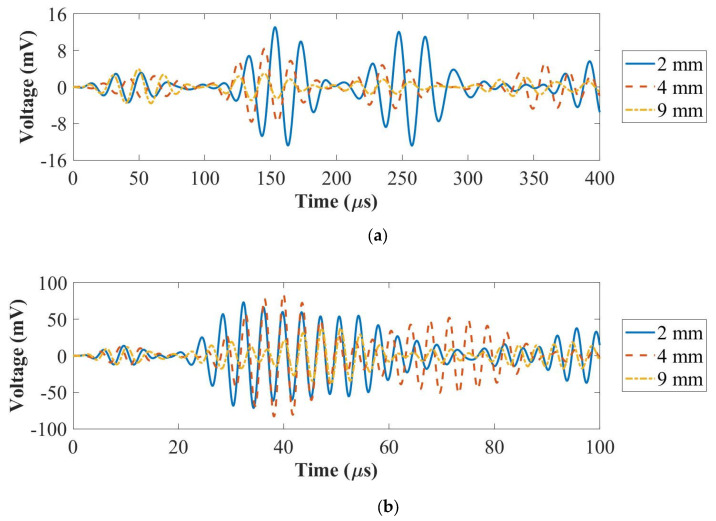
UGW comparisons for the panels with the thicknesses of 2 mm, 4 mm and 9 mm at 50 kHz (**a**) and 250 kHz (**b**).

To compare the amplitude of guided wave signals of each panel, the configuration of path 3–4 with two PZT transducers is shown in Figure 1a. A National Instrument (NI) PXI-5412 arbitrary signal generator and a NI PXI-5105 digitizer were used for signal generation and recording guided wave signals, respectively. The actuation amplitude and the sampling frequency were 6 V and 100 MHz, respectively. Path 3–4 with two PZT transducers is shown in Figure 1a, together with 5 cycles of Hanning-windowed tone-burst signals at 50 kHz and 250 kHz, as shown in Figure 5.

As is shown in Figure 5a, the amplitude of the first wave packet (A_0_ mode) for the 2 mm thick panel was higher than that of the 4 mm and the 9 mm thick panels at 50 kHz. In Figure 5b, the amplitude of the first wave packet for the 2 mm thick panel was lower than that of the 4 mm thick panel and higher than that of the 9 mm thick panel at 250 kHz. In conclusion, the amplitude of the first wave packet for the A_0_ mode reduced with the increased thickness. The group velocity of the A_0_ mode increased with the panel thickness increased from 2 mm to 4 mm and then to 9 mm. In addition, the amplitude of the first wave packet for the S_0_ mode increased first and then reduced. The group velocity of the S_0_ mode decreased when the panel thickness increased from 2 mm to 4 mm and then increased with the increased panel thickness from 4 mm to 9 mm.

**Figure 6:**
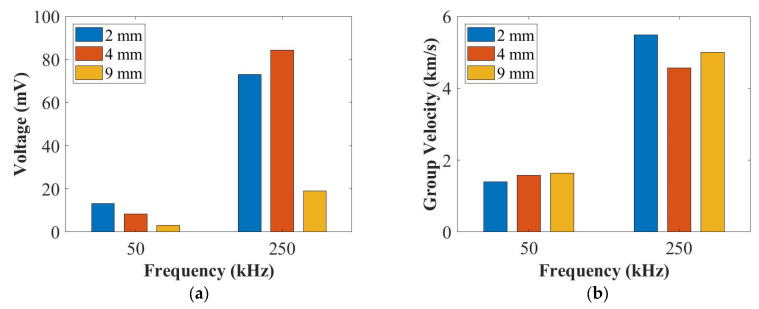
The summary of the amplitudes (**a**) and the group velocities (**b**) of UGWs for the panel thicknesses of 2 mm, 4 mm and 9 mm at 50 kHz and 250 kHz.

Figure 6 summarises the amplitude and the group velocity for the panel thicknesses of 2 mm, 4 mm and 9 mm at 50 kHz (A_0_ mode) and 250 kHz (S_0_ mode). The peak amplitude was computed by choosing the peak amplitude of the measured envelope signals by using the Hilbert transform. The group velocity was computed by the distance between the actuator and the sensor divided by the times of arrival of their envelope signals. As can be seen in Figure 6a, the amplitude of the A_0_ mode was influenced more than that of the S_0_ mode by the thickness. This is because the A_0_ mode was an out-of-plane motion and the S_0_ mode was an in-plane motion. In Figure 6b, the group velocity of the A_0_ mode increased with the increased thickness at 50 kHz, which was affected by the thickness. Furthermore, the group velocity of the S_0_ mode for the 9 mm panel was faster than that of the 4 mm thick panel and slower than that of the 2 mm thick panel, which was not affected by the thickness.

The results presented so far were obtained under room temperature with little variations. Next, the effects of guided wave propagation under varying temperatures in plates with different thicknesses were investigated, which is of high interest for real structures under operational conditions and can affect the reliability of damage detection.

## 5. Temperature Influence on UGWs

**Figure 7:**
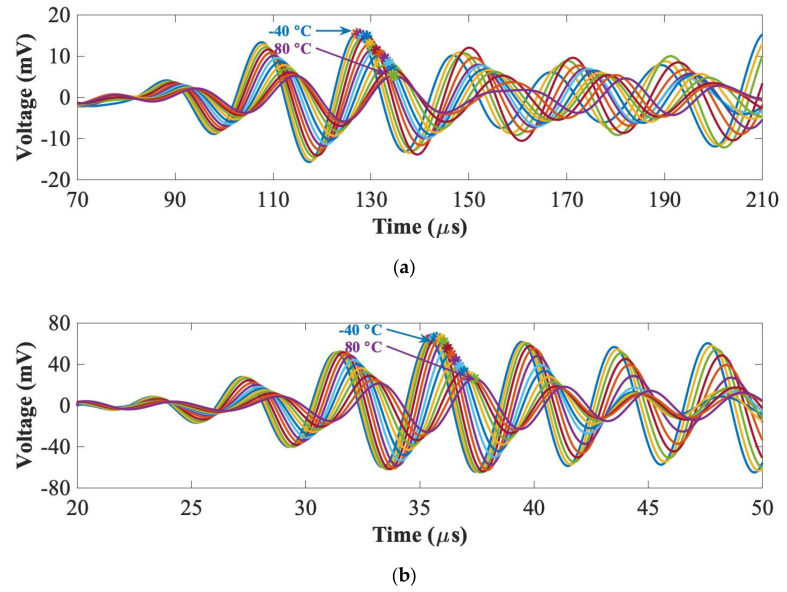
UGWs with peak amplitudes under varying temperatures at 50 kHz (**a**) and 250 kHz (**b**) for the 2 mm thick panel.

Temperature change affects UGWs in two ways: change in phase and change in amplitude, both of which can significantly compromise the reliability of the diagnosis if not compensated. There are several detailed investigations into the effect of the temperature on guided wave signals for think composite plates [42]. However, no research has reported the temperature effect in thick composites and whether there is any dependency on the thickness which then should be taken into account when using temperature compensation algorithms. Therefore, in this section, the temperature effect on the peak amplitude of the first wave packet of UGW signals was evaluated. The three panels were placed into an environmental chamber, and the temperature was varied from −40 °C to 80 °C with a step of 10 °C. The PZT transducer path 3–4 shown in Figure 1a was measured by 5-cycle Hanning-windowed tone-burst signals at 50 kHz and 250 kHz. Figure 7 plots an example of UGWs for the 2 mm thick panel at 50 kHz and 250 kHz. The peak amplitudes of the first wave packets under each temperature were computed automatically to measure the amplitude reduction and the phase shift.

**Figure 8:**
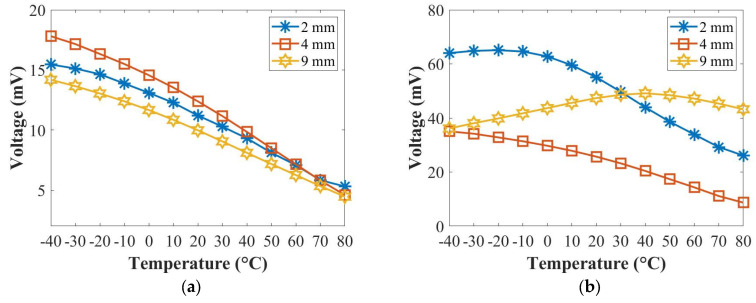
Relationships of the temperature with the peak amplitude at 50 kHz (**a**) and 250 kHz (**b**).

**Figure 9:**
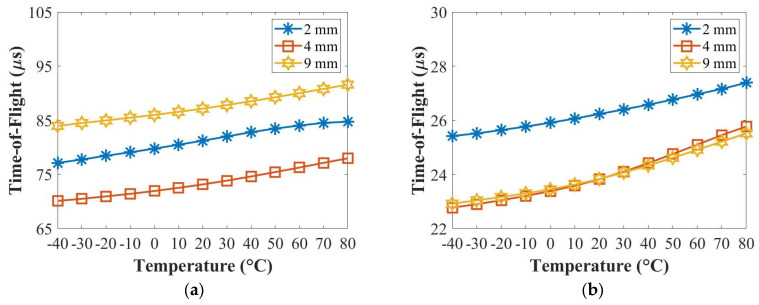
Relationships of the temperature with the time-of-flight at 50 kHz (**a**) and 250 kHz (**b**).

Figure 8 and Figure 9 summarise the relationships of the temperature with the amplitude and the time-of-flight (ToF) at 50 kHz (A_0_ mode) and 250 kHz (S_0_ mode) for the panel thicknesses of 2 mm, 4 mm and 9 mm. The amplitude value was chosen from the peak amplitude of the first wave packet of each signal, and the ToF value was the difference between the time at the peak amplitude of the first wave packet and the half of the actuation signal. In Figure 8a, the amplitudes of the A_0_ mode for the panel thicknesses of 2 mm, 4 mm and 9 mm decreased at 50 kHz. In Figure 8b, the amplitude of the S_0_ mode for the 2 mm thick panel decreased and that for the 4 mm thick panel increased slightly first and decreased, while that for the 9 mm thick panel increased from −40 °C to 40 °C and then decreased from 40 °C to 80 °C at 250 kHz with the increased temperature. In Figure 9a,b, the ToFs of both the A_0_ mode and the S_0_ mode increased for the panel thicknesses of 2 mm, 4 mm and 9 mm with the increased temperature at 50 kHz and 250 kHz.

Overall, the amplitude of the A_0_ mode reduced with the increased temperature for all panels, which was not dependent on thickness, while that of the S_0_ mode had different trends for panels of different thicknesses. In addition, the group velocities became slow for both the A_0_ and the S_0_ modes for all those panels with the increase in temperature, which were not dependent on thickness.

Temperature changes can be attributed to the following factors: First, the piezoelectric constants *d_31_* and *g_31_* vary with the temperature [40,43,44,45]. These piezoelectric constants are associated with the shear strain and the piezo sensitivity, which affects the output amplitude of signals. In addition, thermal expansion also affects changes in the thickness of the panel, wave propagation and material density, which are key parameters to compute dispersion curves under different temperatures [40].

Next, the temperature has a large effect on the resin properties. The resin is more brittle under a very low temperature, while it is softer under a higher temperature. The change in the resin alters the engineering properties of the composite laminate, which causes a change in the shear modulus. The resin interfaces with the carbon, which is sensitive to the temperature and the glass transition temperature.

Furthermore, higher residual strains remain after the curing process inside the laminate when manufacturing the composite laminate. Temperature can affect the residual strains. The efficiencies of the engineering properties could be the residual strains built into the laminate. All of this could affect the temperature. Therefore, it is very important to define the relationship between the phase and the velocity change for composite panels, depending on their thicknesses, so that the compensation algorithm can be applied and damage detection can be carried out under different temperatures reliably.

## 6. Damage Detection and Localisation

In this section, the damage index (DI) correlation coefficient [41] was used to evaluate the damage severities of the structure by comparing the difference between the baseline signals and the current signals. The delay-and-sum (DAS) algorithm [46,47] was used to locate the damage position. To demonstrate the difference between the guided wave interaction with through-thickness damage (i.e., interlaminar delamination due to an impact event) and surface damage (i.e., debonding between two structures), the impact damage and the artificial damage were introduced to the plates with different thicknesses. The surface-mounted artificial damage (weighted Blu Tack) was used in the first instance to simulate the surface **damage** (in this case, it was the increased mass) which was also reversible **before the** plates were impacted to cause barely visible impact damage.

### 6.1. Surface-Mounted Artificial Damage

**Figure 10:**
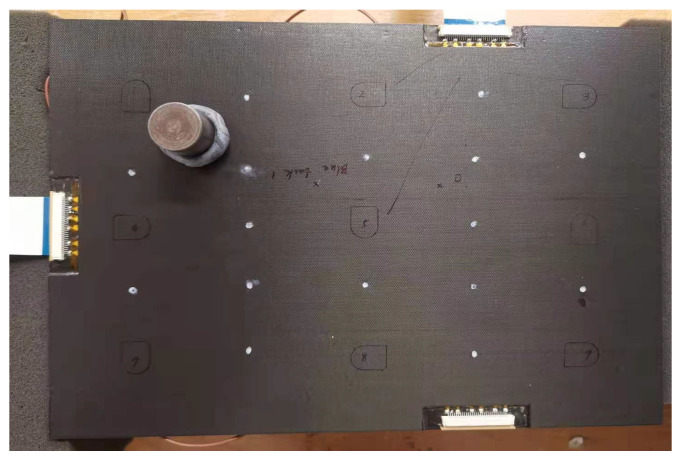
Photo of the surface-mounted artificial damage on the surface of the opposite side of the PZT transducers for the 2 mm thick panel.

In this section, a weighted Blu Tack was closely placed on the surface of the composite panel to simulate the type of surface-attached damage. To verify the thickness effects on damage detection and localisation for the PZT transducers, the Blu Tack was placed on the composite surface of the opposite and the same side (off-centre) of PZT transducers for each panel, and the schematic of the surface-mounted artificial damage (a weighted Blu Tack on the surface of the opposite side of the PZT transducers) can be seen in Figure 10.

**Figure 11:**
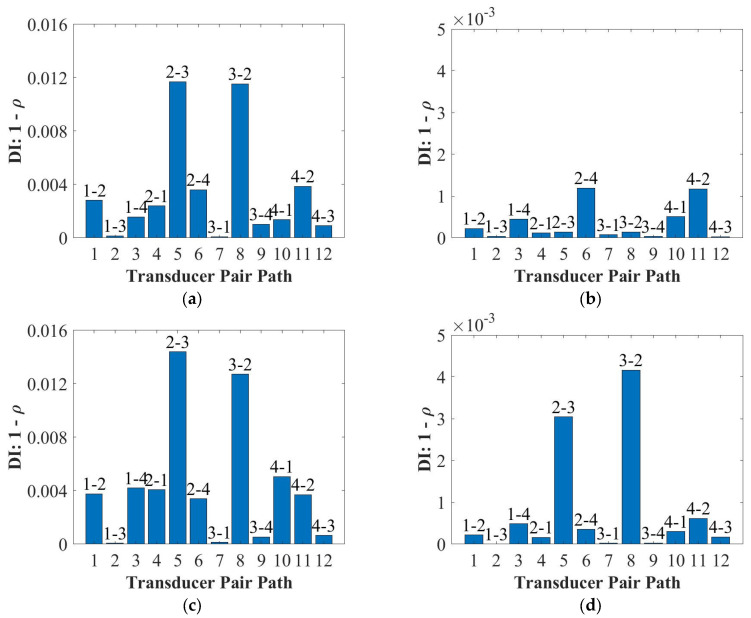
Damage detections for the 9 mm thick panel at 50 kHz (**a**) and 250 kHz (**b**) for the opposite side of the Blu Tack and at 50 kHz (**c**) and 250 kHz (**d**) for the same side of the Blu Tack.

**Figure 12:**
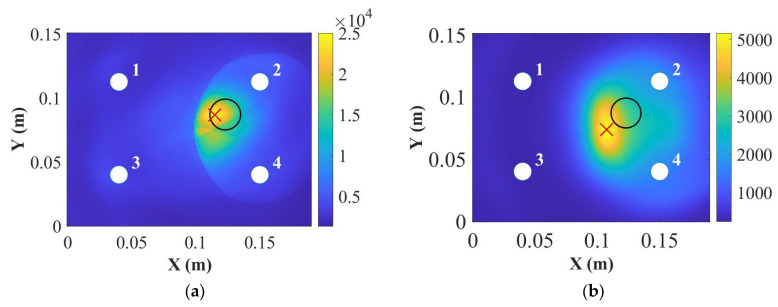

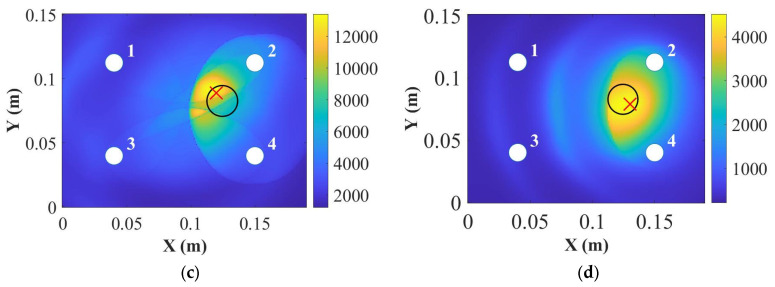
Damage localisations for the 9 mm thick panel at 50 kHz (**a**) and 250 kHz (**b**) for the opposite side of the Blu Tack and at 50 kHz (**c**) and 250 kHz (**d**) for the same side of the Blu Tack (where “**○**” is the position for real damage and “×” is the position for predicted damage).

**Figure A1:**
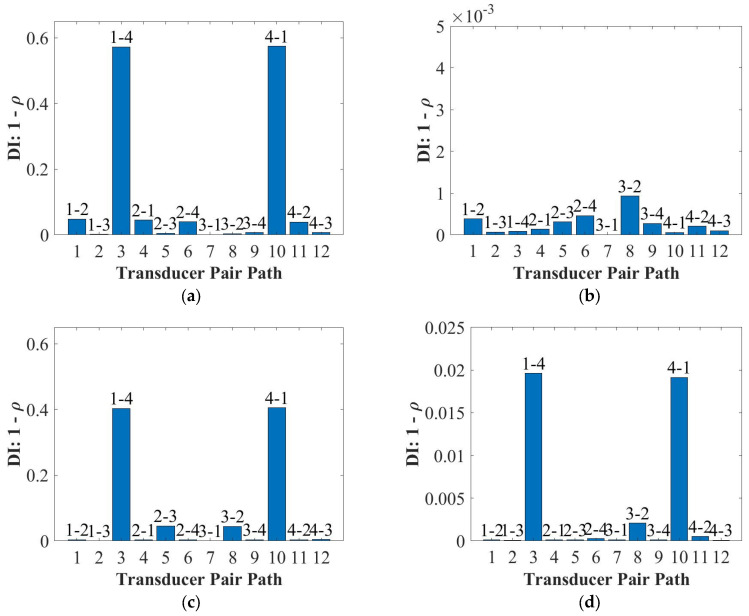
Damage detections for the 2 mm thick panel at 50 kHz (**a**) and 250 kHz (**b**) and for the 4 mm thick panel at 50 kHz (**c**) and 250 kHz (**d**).

**Figure A2:**
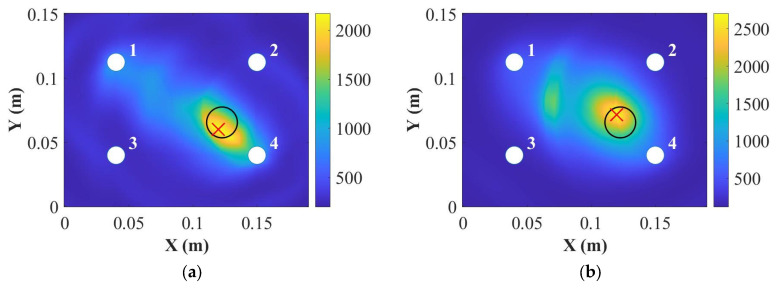

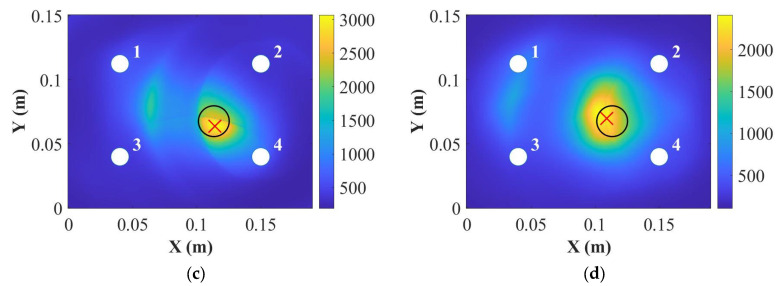
Damage localisations for the 2 mm thick panel at 50 kHz (**a**) and 250 kHz (**b**) and for the 4 mm thick panel at 50 kHz (**c**) and 250 kHz (**d**) (where “○” is the position for real damage and “×” is the position for predicted damage).

Since the DI and the DAS algorithms can accurately detect and locate the surface-mounted artificial damage for the 2 mm and 4 mm thick panels (Figure A1 and Figure A2 in Appendix A), the results for the 9 mm thick panel were reported in this section. Figure 11 and Figure 12 plot the results of damage detections and localisations at 50 kHz and 250 kHz when the damage was closely attached on the opposite and same sides of the PZT transducers for the 9 mm thick panel. As can be seen in Figure 11, the surface-mounted artificial damage (added mass) can be detected by the DI for the panel thickness of the 9 mm, when the weighted Blu Tack was attached on both the opposite and same sides of the PZT transducers. Meanwhile, the DI results showed that the A_0_ mode had a better detection of the surface type of damage (weighted Blu Tack) than the S_0_ mode.

In addition, the DAS algorithm showed this type of damage cannot be located accurately for the S_0_ mode in the 9 mm thick panel at 250 kHz shown in Figure 12b. These can be related to the dispersion curve shown in Figure 4. The group velocities of the A_0_ and the S_0_ modes were closer, which may affect the results of damage localisation. However, when the surface-mounted artificial damage was attached on the same side of the PZT transducers, the damage was located accurately at 250 kHz for the 9 mm thick panel, as shown in Figure 12d. This may be because the S_0_ mode was less sensitive for thick composites, so that the accuracy of the DAS algorithm was affected. Next, the panels were impacted to cause damage, and the appropriateness of the different modes for detection was investigated.

### 6.2. Impact Damage

**Figure 13:**
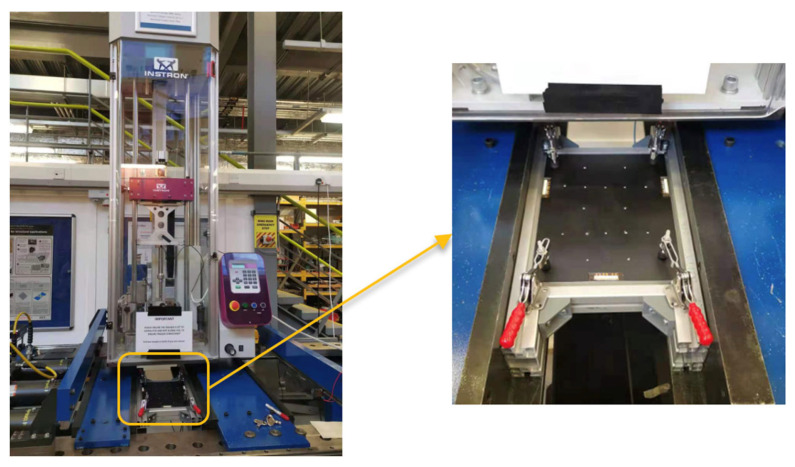
INSTRON CEAST 9350 drop tower for the impact test.

To study the real impact damage, impact tests were conducted on the above 2 mm, 4 mm and 9 mm thick panels. Barely visible impact damage (BVID) was considered in these tests. A drop tower INSTRON CEAST 9350 was used for these impact tests, as is shown in Figure 13. The impact position for each panel was on the opposite side of the PZT transducers and was the same as one of the positions for the surface-mounted artificial damage. The impact energy started from a low level and then increased step by step until a suitable damage area was detected by a DolphiCam C-scan (CF08). The hemisphere diameter of the impactor was 20 mm. For the 2 mm thick panel, a 22 kN load cell was used with a 3 kg additional mass. The impact energy was 15 J, and the impact velocity was 3.53 m/s. For the 4 mm and 9 mm thick panels, a 90 kN load cell was used with a 3 kg additional mass. The impact energy and velocity were 20 J and 2.72 m/s for the 4 mm thick panel, respectively, and were 57 J and 4.59 m/s for the 9 mm thick panel, respectively. After the impact, the surface cracks were visible on these panels.

**Figure 14:**
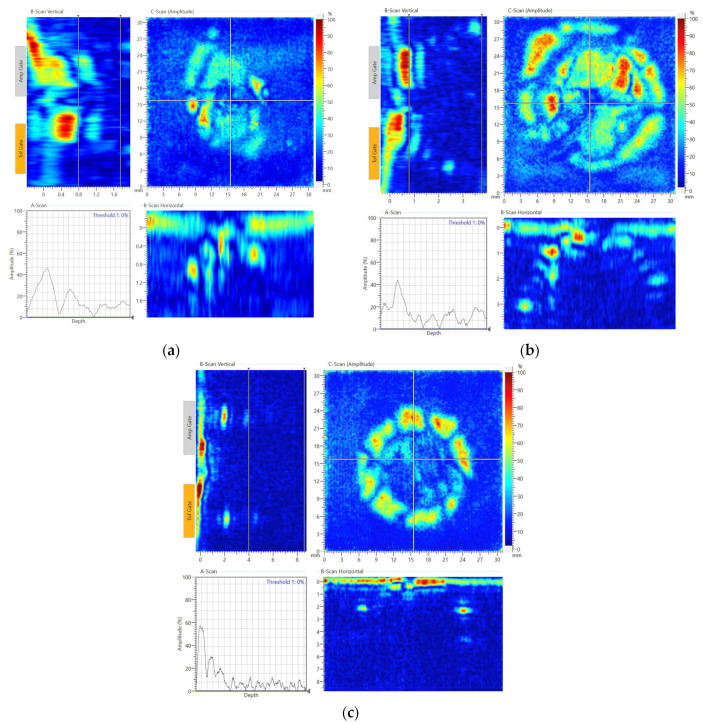
The C-scan results of the impact damage at 15 J for the 2 mm thick panel (**a**), 20 J for the 4 mm thick panel (**b**) and 57 J for the 9 mm thick panel (**c**).

Figure 14 shows C-scan results for the impact damage of each panel. In Figure 14a, the detected damage area was about 176 m^2^, and the delamination occurred from the 9th ply to the top surface according to the horizontal and vertical B-scans for the 2 mm thick panel. In Figure 14b, the detected damage area was about 452 mm^2^ for the 4 mm thick panel, and the damage was from the 24th layer to the top surface according to the horizontal and vertical B-scans. As shown in Figure 14c, the detected damage area was about 254 mm^2^ for the 9 mm thick panel. The delamination occurred from the 8th layer to the top surface according to the horizontal and vertical B-scans.

**Figure 15:**
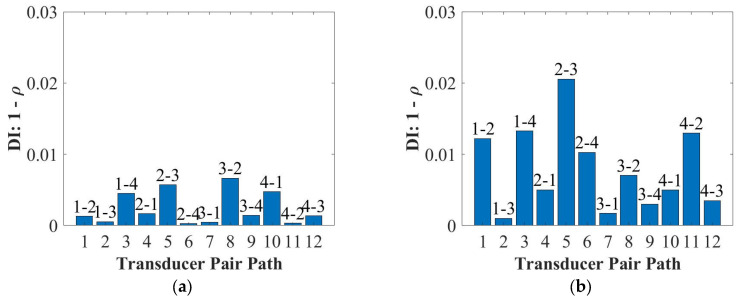
Damage detections for the 9 mm thick panel at 50 kHz (**a**) and 250 kHz (**b**).

**Figure 16:**
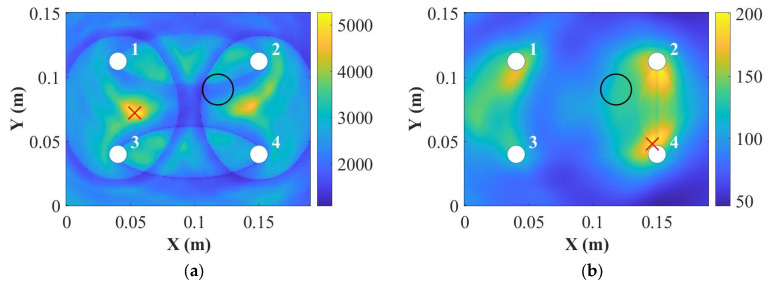
Damage localisations for the 9 mm thick panel at 50 kHz (**a**) and 250 kHz (**b**) (where “**○**” is the position for real damage and “×” is the position for predicted damage).

In this section, the results of detection and localisation of the impact damage at 50 kHz and 250 kHz for the 9 mm thick panel were shown in Figure 15 and Figure 16, respectively. As can be seen in Figure 15 and Figure 16, the damage index can detect the impact damage, but the DAS algorithm cannot locate the impact damage accurately.

For the 9 mm thick panel, neither of the frequencies showed robust and reliable results of detection and localisation, which is attributed to the small severity of the damage as shown in Figure 14c. The extent of damage can be hardly noticed in the C-scan through the thickness; therefore, the damage reflected waves were of small amplitude and fell mainly within the noise level (very small DI values). Therefore, the damage should be extended for reliable detection. Another important factor is that for the delay-and-sum results usually guided waves are chosen in their non-dispersive zones. As can be seen in Figure 14b, the thickness greatly affected the group velocity of the S_0_ mode at 250 kHz. In addition, the group velocity for the 9 mm thick plate at 250 kHz was dispersive, which caused the S_0_ and the A_0_ modes to interact with each other. Therefore, the localisation results were not accurate as was also noticed here.

## 7. Conclusions

This paper studied the influence of the composite thickness on guided wave signals under varying temperatures and resulting damage detection. The results of dispersion curves showed that the thickness significantly affected the group velocity of the S_0_ mode at 250 kHz. In addition, it has been found that the amplitude of the A_0_ mode was influenced more than that of the S_0_ mode by the thickness variation. In addition, the group velocity of the A_0_ mode increased with the increased thickness, while the group velocity of the S_0_ mode for the 9 mm thick panel was faster than that of the 4 mm thick panel and slower than that of the 2 mm thick panel. This was attributed to the dispersive nature of the S_0_ mode at the frequency/thickness-combined effect for the 9 mm thick panel at 250 kHz.

Overall, the amplitude of the A_0_ mode was not dependent on the thickness as it reduced with the increase of the temperature for all panels, while that of the S_0_ mode had different trends for panels with different thicknesses. In addition, the group velocities became slower for both the A_0_ and the S_0_ modes for all those panels with the increase of the temperature, which was not dependent on the thickness. Therefore, it is very important to define the relationship between the phase and the velocity change for composite panels, depending on their thicknesses, so that the compensation algorithm can be applied and damage detection can be carried out under different temperatures reliably.

The DI results showed that the A_0_ mode had a better detection of surface-mounted artificial damage (weighted Blu Tack) than the S_0_ mode. The DAS algorithm showed this type of damage could not locate accurately for the S_0_ mode in the 9 mm thick panel at 250 kHz when the weighted Blu Tack was attached on the opposite side of the PZT transducers. However, the DAS algorithm could locate the damage accurately at 250 kHz for the 9 mm thick panel, when the surface-mounted artificial damage was attached on the same side of the PZT transducers. Hence, the S_0_ mode became less sensitive, when the thickness of the composite panel increased.

Then, the guided wave propagation and response with PZTs for the detection of impact damage were studied. For the 9 mm thick panel, neither of the frequencies showed robust and reliable results in detection and localisation, which was attributed to the small severity of the damage in the C-scan result (hardly noticed along the thickness direction). Therefore, the damaged reflected waves were of small amplitude and fell mainly within the noise level, and the damage should be extended for reliable detection. Another important factor was that for delay-and-sum results, guided waves are usually chosen in their non-dispersive zones, but the result of the dispersion curves for the 9 mm thick panel at 250 kHz showed that the group velocities for the A_0_ and the S_0_ modes were too close to be distinguished from each other and the localisation results were not accurate.

## Data Availability

Not applicable.

## Appendix A

In this section, the surface-mounted artificial damage was closely placed on the opposite surface of the PZT transducers for the 2 mm and 4 mm thick panels. Figure A1 and Figure A2 plot the results of damage detections and localisations at 50 kHz and 250 kHz. As can be seen in these figures, the surface-mounted artificial damage (added mass) can be detected by the DI for the panel thicknesses of 2 mm and 4 mm. Meanwhile, the DI results showed that the A_0_ mode had a better detection of the surface type of damage (Blu Tack) for the 2 mm and 4 mm thick panels. In addition, the DAS algorithm shows this type of damage can be located accurately at 50 kHz and 250 kHz.
